# Comparative RNA sequencing-based transcriptome profiling of *Quercur robur*: specific sets of genes involved in taproot and lateral roots emergence

**DOI:** 10.1093/treephys/tpaf067

**Published:** 2025-06-02

**Authors:** Paulina Kościelniak, Paulina Glazińska, Agnieszka Bagniewska-Zadworna, Joanna Mucha, Marcin Zadworny

**Affiliations:** Faculty of Biology, Institute of Human Biology and Evolution, Adam Mickiewicz University, Uniwersytetu Poznańskiego 6, 61-614 Poznań, Poland; Faculty of Biological and Veterinary Sciences, Department of Plant Physiology and Biotechnology, Nicolaus Copernicus University, Lwowska 1, Toruń 87-100, Poland; Faculty of Biology, Department of General Botany, Institute of Experimental Biology, Adam Mickiewicz University, Uniwersytetu Poznańskiego 6, 61-614 Poznań, Poland; Department of Ecology, Institute of Dendrology, Polish Academy of Sciences, Parkowa 5, Kórnik 62-035, Poland; Faculty of Forestry and Wood Technology, Poznan University of Life Sciences, Wojska Polskiego 71c, Poznań 60-625, Poland; Department of Ecology, Institute of Dendrology, Polish Academy of Sciences, Parkowa 5, Kórnik 62-035, Poland

**Keywords:** gene expression, oak, RNA-Seq, roots growth

## Abstract

Root development is well recognized in model plants, with many studies focusing only on primary root growth or lateral root initiation. However, taproot vs lateral root development has rarely been explored using molecular tools, and even less is understood about how the molecular processes engaged in taproot elongation shape the emergence of lateral roots in trees in the time-dependent manner. We address how gene expression is associated with elongation of taproot and lateral root formation of *Quercus robur* L. In addition, we have analyzed how the exogenous application of hormones and inhibitors shapes the root architecture. We also revealed that lateral root formation and emergence corresponds to expression of genes at specific taproot length points. Therefore, our study suggests that the pattern of gene expression in the taproot tips is involved in the shaping of lateral root growth. In addition, we have shown that lateral roots are characterized by a set of genes that are distinct from those expressed in the taproot tips. Insights from this study contribute to better understanding root development in trees.

## Introduction

Root systems’ exploration of diverse soil environments is a function of internal and external factors that jointly control long primary root (i.e., taproots, TR) formation and further lateral root growth (Petricka et al. 2012). Although empirical evidence suggest that processes contributing to the two root formations may be different, it is still unclear whether taproot and lateral root growth are similarly controlled by the same signaling pathways, as straightforward long-distance growth toward deep soil layers of the primary roots (i.e., the first root emerging from the seed) should already be mediated at the embryonic stage to: (i) maintain a fast growth rate and (ii) regulate root architecture patterning. On the other hand, lateral roots (synonyms: secondary root or branch root) arise as branches from the pericycle of the taproot, and the mechanism of their initiation and further growth is not only environmentally responsive but may also be controlled by the taproot (Desnos 2008, Lavenus et al. 2013). The processes driving primary and lateral root formation may be universal or remarkably specific, and root type development can be linked to plant persistence in the environment (annual or perennial). Indeed, a regulatory signaling network of primary and lateral root formation has been generated for Arabidopsis, characterized by uncomplicated cellular organization, cannot be easily extrapolated to other plants with distinct ontogenic programs, such as long-lived oaks (Lavenus et al. 2015, Jeon et al. 2021). Therefore, root development in perennial plants requires an integrated assessment to identify how primary roots (i.e., taproots) growth is modulated after emergence when roots become longer and roots as a whole have to respond to environmental changes along the soil gradient (Jin et al. 2013). Thus, despite the importance of mechanisms underlying taproot and lateral root growth, our understanding of how relative contribution of endogenous factors is involved in taproot growth and lateral roots initiation is insufficient.

Initiation of taproot growth and further elongation across soil had profound consequences for root system architecture, involving varied gene regulatory networks, transcription factors and hormone distribution engagement (Scheres et al. 2004, Du and Scheres 2017, Wendrich et al. 2017, Svolacchia et al. 2020). Identifying and understanding the functions of genes promoting root elongation and growth in trees, which may have to activate and silence various genes and transduce cross-root zone signals many times, can provide valuable information regarding the factors and signal cascades playing central roles in root growth (Casson and Lindsey 2003, Chaiwanon et al. 2016, Slovak et al. 2016). Genetic, transcriptional and hormonal factors coordinate not only deep root growth (e.g., primary roots) but also branching capacity of laterals (Casson and Lindsey 2003, Xuan et al. 2016). The influence of gene interplay is especially evident during the modulation of root growth under water shortage (Carlsbecker et al. 2010, Haswell and Verslues 2015, Müller et al. 2016), where it has been demonstrated that the stimulation or inhibition of primary root growth is controlled through regulation of phosphate-isopentenyltransferase expression affecting cytokinin (CK) content (Nishiyama et al. 2011). Moreover, the sequential stages of primary root growth regulated by transcription factors (TFs) (Sarkar et al. 2007, Drisch and Stahl 2015, Mitsis et al. 2020, Kościelniak et al. 2021, Kościelniak et al. 2024) and TFs driving auxin (indole-3-acetic acid—IAA) transport may regulate other auxin response genes (Weijers et al. 2006). These, together with WOX TFs (WOX 5/7 and WOX11), not only induce and sustain primary root growth but also regulate lateral root development arising from primary roots (Hu and Xu 2016, Baesso et al. 2018). It has also been shown that an increase in *PINs* expression contributes to the initiation of primary root development, but PINs are not entirely responsible for the arrangement of lateral root elongation (Blilou et al. 2005, Vieten et al. 2005), confirming that characterizing the genetic basis of signals promoting root growth enables understanding of functional differences in pathways governing divergent root growth. An analysis of PIN2 factors indicated a restriction of lateral but not primary root growth by increasing its sensitivity to auxin transport, enhanced by gibberellins signaling (Gou et al. 2010, Li et al. 2018, Waidmann et al. 2019). These studies suggest that the synergy of gene expression may coordinate the growth of both root types and regulates primary root growth orientation into wet areas located deeper in the soil profile (Pierret et al. 2016). However, the mechanism of gene expression patterns during taproot elongation and the specific role of its meristem in lateral root initiation and growth maintenance (Atkinson and Halfon 2014) remains an open question in trees. The challenge is to assess how growth patterns enabling taproots to reach deep soil layers are regulated in trees and whether the molecular regulatory mechanisms involved in the growth of primary roots are also involved during the formation of lateral roots, as there is limited evidence for the same transcriptomic pattern among both root types in perennial plants. It has been confirmed that dynamic soil exploration results from transcriptional signaling networks (Waidmann et al. 2019). Thus, a plant’s ability to diversely modulate primary and lateral roots, whether it persists for one or many years, may be of special importance for shaping taproot system architecture (Smith and De Smet 2012) and may be attributed to the gene expression pattern during taproot growth. Therefore, the need for gene expression network remodeling in relation to a plant’s life strategy must be addressed because genes may operate differently in annual herbaceous species and trees at different stages of root growth.

The aim of our study was to evaluate the temporal changes in gene expression patterns during taproot and lateral root elongation and determining changes in the level of these factors between the taproot and the lateral root. We determined: (i) interconnection between lateral roots emergence and specific length of taproot; and (ii) upregulation of gene involved in lateral roots initiation by signals from taproot. To achieve this goal, we performed RNA sequencing (RNA-Seq) to obtain a complete picture of the taproot and lateral roots transcriptome. Expression analysis of differentially expressed hormone-associated genes and genes coding transcription factors allowed us to identify potential differences in the expression profiles of specific hormones and changes in the activity of genes encoding elements of signal transduction pathways, hormonal responses and key transcription factors depending on the size of taproots and lateral roots. We report that differential gene expression patterns during root growth improve our understanding of taproot and lateral root development at a genetic expression cascade in long-lived trees, providing supportive evidence for our hypothesis that taproot regulators play a significant role in shaping the growth of oak roots. Additionally, to confirm the role of hormones whose coding genes were identified in the RNA-Seq analyses, we conducted hydroponic experiments using exogenous hormones to validate their impact on root growth and development.

## Materials and methods

### Plant material cultivation and sample collection

**Figure 1:**
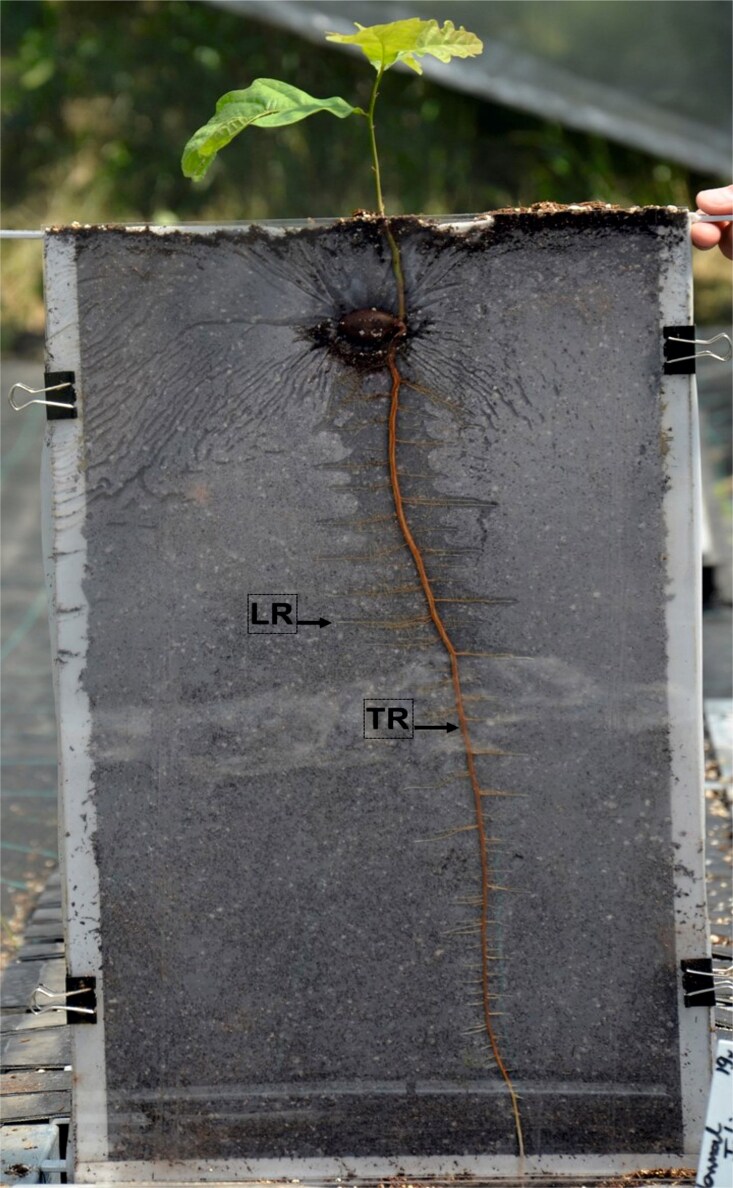
Sample image of well-established *Quercus robur* seedling growing in rhizotron system. TR—taproot, LR—lateral roots.

**Figure 2:**
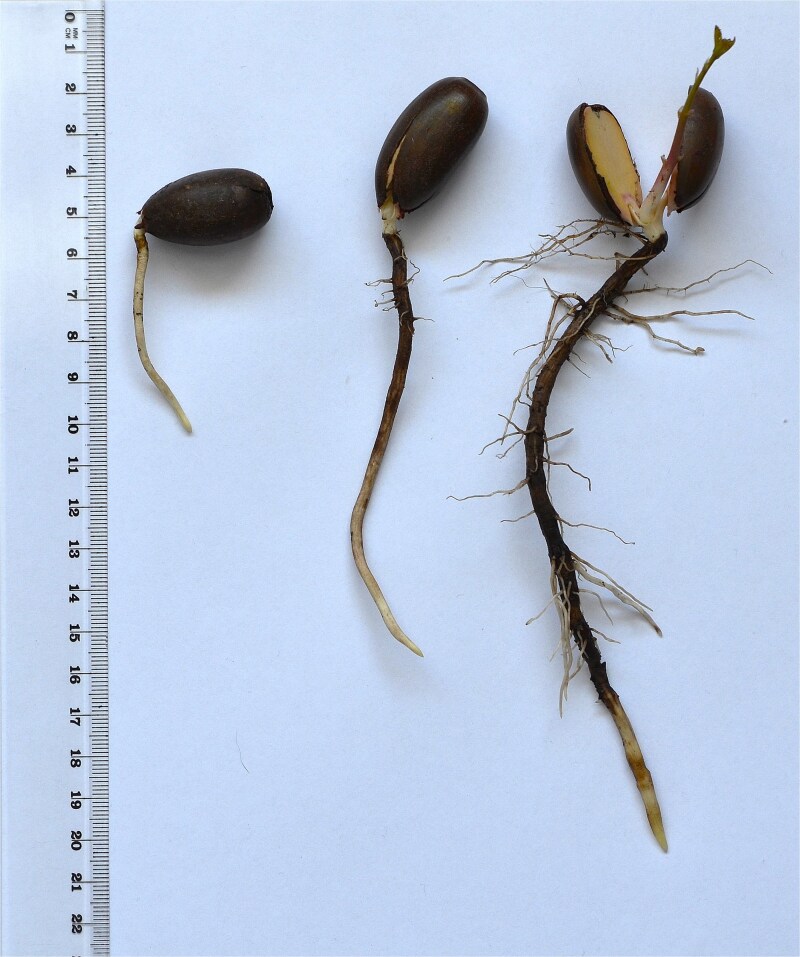
The morphology of *Quercus robur L.* taproots among short (left), medium (central) and long (right) length classes.

**Figure 3:**
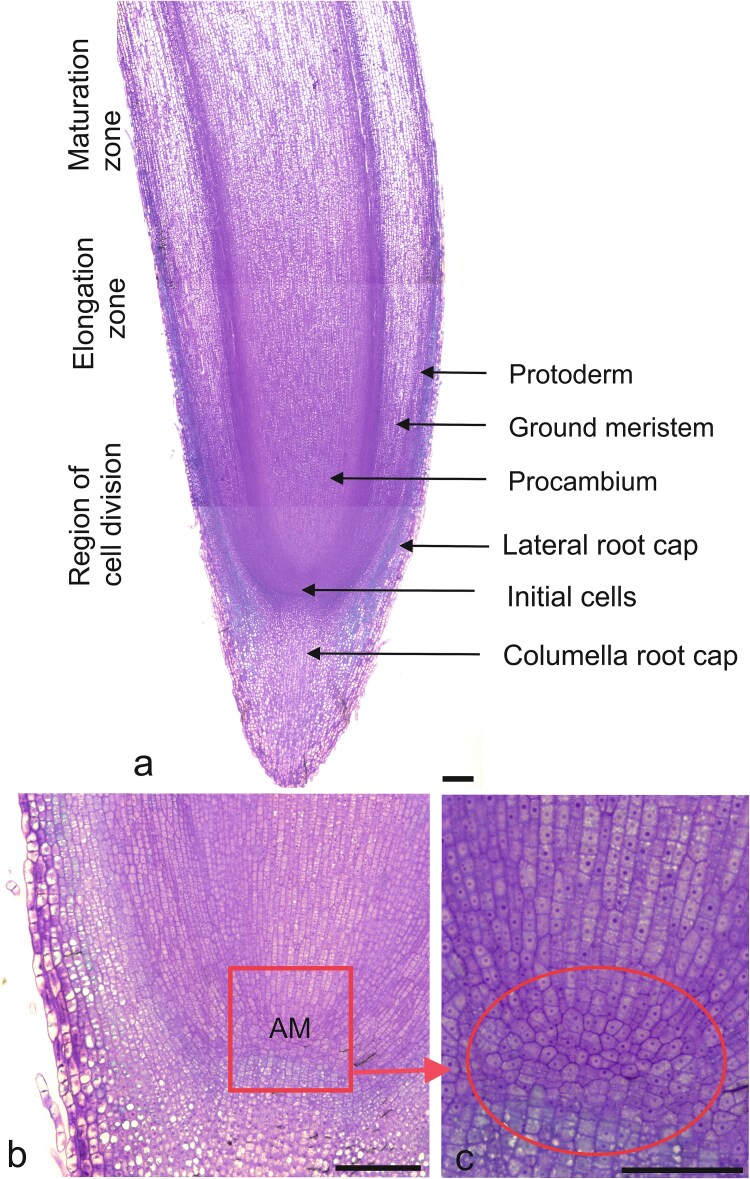
The longitudinal sections of *Quercus robur* taproot tips illustrating different root growth zones and apical meristem (AM). Scale bars: (a, b) = 200 μm, (c) = 100 μm.

The experiment took place in a large, semi-closed for foil greenhouse situated in the Institute of Dendrology, Polish Academy of Sciences, Kórnik, Poland. The 4-m-high roof of the greenhouse protected seedlings from rainfall, while open ends and a 0.5-m height opening in the lateral walls provided sufficient ventilation for temperatures inside the greenhouse to remain close to ambient temperatures in the surrounding environment, and mean average temperature is 15.3 °C for the experiment period. After the rhizotrons were embedded in the ground, the average soil temperature at 0–20 cm depth was 14.2 °C throughout the experiment. The experiment was conducted in a clear-walled rhizotron chamber (30 × 50 cm), filled with a growing medium of peat and perlite (proportions 5:1 volumetric proportion), deacidified with dolomite and enriched with 2.5 kg m^−3^ slow-release fertilizer (Osmocote 15-9-12-2 N-P-K-Mg, with trace nutrients). Please see the illustrative photo (Figure 1). Drainage in the bottom of each rhizotron protected from waterlogging. Acorns from *Quercus robur* L. (*Q.robur*) of the same genetic origin/provenance (seeds were harvested from the same area at the same time) were sown directly in the rhizotron during the spring. Each taproot was classified at its time of harvest based on its length: short (5–9 cm), medium (9.5–15 cm) and long (>15.5 cm), and marked as short taproot characterized by standard morphology (STR) for short, medium taproot characterized by standard morphology (MTR) for medium and long taproot characterized by standard morphology (LTR) for long, respectively. Although the acorns were sown simultaneously, their germination timelines varied. As a result, the plants exhibited different root lengths: shorter roots were younger than medium roots, and medium roots were younger than long roots. At the time of harvest, we removed the plastic wall to expose the root system. We then measured the diameter of the root tips and the proximal part (base) of STR, MTR and LTR. The root system of the seedlings was then gently washed with deionized, autoclaved water (ddH_2_O) to remove adhering soil, and then the 5–6 mm long root tips containing the meristematic zone of the taproots or their lateral were dissected, immediately frozen in liquid nitrogen and stored at −80 °C until RNA extraction (Figure 2). To confirm that the excised tip contained the meristematic zone was confirmed by histological analyses (see Figure 3). For each biological replicate, we pooled 10 taproot tips from separate plants or 50 lateral root tips collected from the same 10 roots. This resulted in one pooled sample per replicate for RNA-Seq analysis. We used STR, MTR and LTR for anatomical analyses, and MTR and LTR with their lateral roots for RNA-Seq analyses. Three biological replications were used for RNA-Seq analyses for every type and length of roots.

### RNA isolation and sequencing analysis

RNA was isolated from the ground material using Ribospin (GeneAll Biotechnology in Seoul, South Korea). Subsequently, a cDNA library was created using a TruSeq Stranded mRNA LT Sample Prep Kit and the library was sequenced using a NovaSeq platform from Illumina (San Diego, CA, USA) in the 150 bp paired-end mode. The assembled transcripts were mapped to the reference genome. Reads were mapped against a *Q. robur* genome Qrob_PM1N.fa (https://urgi.versailles.inra.fr/download/oak/Qrob_PM1N.fa.gz) using STAR 2.5.3a. Annotation of the transcriptome was performed with Trinotate v 3.0.2. This was done in several steps: (i) a search against Swiss-Prot proteins, a nonredundant and manually curated set of proteins from UniProt database. Here, BLASTX from BLAST package, with -max_target_seqs 1 option was used; (ii) open reading frames were predicted with TransDecoder v 5.0.1 and the predicted protein sequences were searched against Swiss-Prot using BLASTP from BLAST package; (iii) the transcriptomes were searched against protein domains from Pfam database using hmmscan with default settings and were loaded into an sqlite database of Trinotate. Identification of TFs was accomplished using PlantTFcat with default settings. The prediction was done for protein sequences predicted with TransDecoder using an online interface available at http://plantgrn.noble.org/PlantTFcat/. The information presented in this publication has been stored in NCBI GEO and can be accessed through the GEO Series accession number GSE181860.

### Identification of differentially expressed genes

Expression values were obtained with RNA-Seq by Expectation-Maximization (RSEM) using Bowtie 2 as a mapper and observing the reads strandedness (Langmead and Salzberg 2012). Gene and single transcript expression levels were analyzed and presented in FPKM (Fragmentst Per Kilobase Of Exon Per Million Fragments Mapped). The expected count represents the number of paired reads that were mapped to a specific gene/transcript. Differential analysis was performed with DESeq2 at the transcript level (Love et al. 2014). Genes whose expression changed at least 1.5 times (fold change > 1.5) with a statistical probability cut-off *P*-value < 0.05 were considered to be differentially expressed. The Transcript IDs listed in the Table S1 available as Supplementary Data at *TreePhysiology* Online display various isoforms of the genes mentioned in the article, and their differences in expression can be checked by entering the Transcript ID in the OakRootRNADB Database https://oakrootrnadb.idpan.poznan.pl/ (Kościelniak et al. 2022). Annotations were made to databases such as Pfam, Pfam GO, BLASTP, BLASTX, BLAST GO and Kyoto Encyclopedia of Genes and Genomes (KEGG).

### Analysis of KEGG and GO

The analysis of GO (Gene Ontology) term overrepresentation was performed using the script from the Trinotate-Trinotate-v3.2.2 package. The GO analysis was conducted at no_ancestral level (gene-level analysis not taking into account the ancestral terms). Obsolete terms were updated using data from the AmiGO database (amigo.geneontology.org). All identified genes for *Q. robur* obtained with Trinotate v 3.0.2 were used as background. Expression values for each comparison were taken from the results of the edgeR program. Only records that contained an enrichment level >1.5 were selected as relevant. The same analysis was performed for the identified KEGG pathways. The results were additionally filtered to include only terms derived from plants.

### Validation of gene expression by RT-qPCR

For the synthesis of first-strand cDNA, 1 μg of total RNA was used as the template, and the process was carried out with SuperScript III Reverse Transcriptase (Invitrogen, Carlsbad, CA, USA) following the manufacturer’s instructions. The resulting cDNA samples w ere diluted fivefold before being used in reverse transcription-quantitative polymerase chain reaction (RT-qPCR). The RT-qPCRs were performed using the SensiFAST Probe No-ROX Kit (Bioline, UK) in accordance with the manufacturer’s guidelines. Each reaction mixture consisted of 2× SensiFAST qPCR Master Mix, forward and reverse primers, a specific Universal Probe Library (UPL) probe, and the diluted cDNA. The reactions were run on a LightCycler480 instrument (Roche, Switzerland) with the following cycling conditions: an initial denaturation at 95 °C for 10 min, followed by 45 cycles of 95 °C for 10 s, 58 °C for 30 s and 72 °C for 1 s. Each gene in the sample groups was analyzed with two biological replicates and three technical replicates. A references gene, *Ubiquitin*, was employed for normalization. The primers and UPL probes were designed using the Universal Probe Library Assay Design Center (Roche, Switzerland), and their details are provided in Table S2 available as Supplementary Data at *TreePhysiology* Online. The LightCycler480 software (Roche, Switzerland) was used to calculate the expression levels from the RT-qPCR data.

### Hydroponic analysis

The mini-hydroponic system was employed to investigate the impact of hormone on seedling growth. The hydroponic arrangement comprised a 10 L opaque plastic container, equipped with a lid featuring perforations and air distributors, connected to an air pump via a non-return valve. Acorns were germinated in autoclaved sand for 21 days, followed by a rinse with sterile water. Thereafter acorns with similar taproot size were transplanted onto the lid of the plastic container, and their roots were immersed in a half-strength Hoagland’s nutrient solution supplemented with increasing concentration IAA inhibitor N-1-naphthylphthalamic acid (NPA), cytokinin-trans-Zeatin (tZ), 6-Benzylaminopurine (BAP), abscisic acid (ABA) and precursor of ethylene 1-Aminocyclopropane-1-carboxylic acid (ACC). The pH of the medium was set at 5.8. We carried out this experiment in an environmental chamber under light emitted by fluorescent tubes (Osram L36/W77 Flora; 100 μEm^−2^ s^−1^) 16 h a day, 60% relative humidity at 20:18 °C day to night temperature ratio. Control plants consisted of seedlings of the same age treated in the same way but without phytohormone supplementation. Each container constituted one biological replication, with three replications per treatment, and 10 seedlings per biological replication were analyzed. Following 3 weeks of growth, we gently removed seedlings from the system. Each harvested plant was separated into leaves, stems, and roots. Roots were scanned fresh using a Perfection 3200 PHOTO scanner (Epson), analyzed using WinRHIZO software (Regent Instruments) to measure root system length (cm), taproot length (cm), root length within given diameter categories (0–0.2 mm, 0.2–0.4 mm, 0.4–0.6 mm, 0.6–0.8 mm, 0.8–1 mm, 1–1.2 mm, 1.2–1.4 mm, 1.4–1.6 mm, 1.6–1.8 mm, 1.8–2 mm), number of fine root tips per root system and mean fresh root diameter (mm), and then roots were dried for 3 days at 65 °C and weighed to enable the determination of SRL (fresh root length per unit root dry mass), RTD (root dry mass per unit fresh root volume) and SRA (fresh root area per unit of root dry mass). Preliminary studies indicate that the substance used to dissolve the hormones has no impact on the analyzed root system’s traits. The main effects of hormones were determined. Differences between mean values across different concentrations of the specific hormone in relation to control were also analyzed using a one-way ANOVA and then a post-hoc Tukey’s HSD test. Statistical relationships were considered significant at *P* ≤ 0.05. All analyses were conducted using JMP Pro 13.

### Anatomical analysis

Simultaneously with the collection of material for RNA-Seq analyses, root segments were excised from STR, MTR and LTR. Six root segments from root meristem, region of elongation and region of maturation were selected from each of the three taproot length classes (18 roots of each root region; 54 root segments in total). The samples were fixed immediately in a solution containing 2% formaldehyde and 2% glutaraldehyde in 0.05 M phosphate-buffered saline, following the protocol outlined by Bagniewska-Zadworna et al. (2012). After a 24-h fixation period, the roots were washed twice in 0.01 M phosphate-buffered saline and then in deionized water twice. The roots were dehydrated in a series of progressively increasing concentrations of ethyl alcohol (10%, 30%, 50%, 70%, 90%, 96% and 100%) for 1 h at each concentration, using ethyl alcohol obtained from Polish Chemical Reagents in Gliwice, Poland. The root samples were subsequently infiltrated with and embedded in Technovit 7100, a resin made by Heraeus Kulzer in Wehrheim, Germany. Cross-sections of roots with a thickness of 5 μm were prepared from the embedded samples using a rotary microtome (Leica RM2265), stained with a solution of 0.1% toluidine blue (Sigma, St Louis, USA) dissolved in 1% sodium tetraborate (also from Sigma, St Louis, USA) or using methylene blue-azure II-basic fuchsin staining as described by Harrison et al. (1974). Cross-sections were observed at 5–40× magnification using a Carl Zeiss Axioskop 20 light microscope (Carl Zeiss, Germany), and photographs were taken using an Axiocam with AxioVision software, also from Carl Zeiss.

## Results

### Taproot morphology

Generally, there were no significant differences in the diameter of root tips among STR, MTR and LTR (1.83 mm, 1.79 mm and 1.42 mm, respectively; *P* = 0.253). In contrast, the diameter at the root base showed an opposite trend, with LTR being wider and MTR and LTR taproots being thinner (3.21 mm vs 2.42 mm vs 2.47 mm, respectively; *P* = 0.044). The occurrence of lateral roots varied with root length class, with a visible lack of lateral roots in short taproots, rare emergence in medium taproots, and abundance in long taproots (Figure 2).

### Taproot anatomy

The tips of STR, MTR and LTR did not differ in their anatomy and tissue organization. Root apical meristems were characterized by an open organization in its apex, which is the region at the tip of a plant root that contains undifferentiated, actively dividing cells responsible for root growth. The tip differentiating cells consisted of three primary meristems: protoderm, which gives rise to the rhizodermis and originates from the outer layer of meristematic cells; ground meristem which develops into the cortex and endodermis (middle layers of the root derived from cells surrounding the vascular tissues); as well as procambium which forms the vascular tissues (xylem and phloem) and is located at the central region of the root (Figure 3).

**Figure 4:**
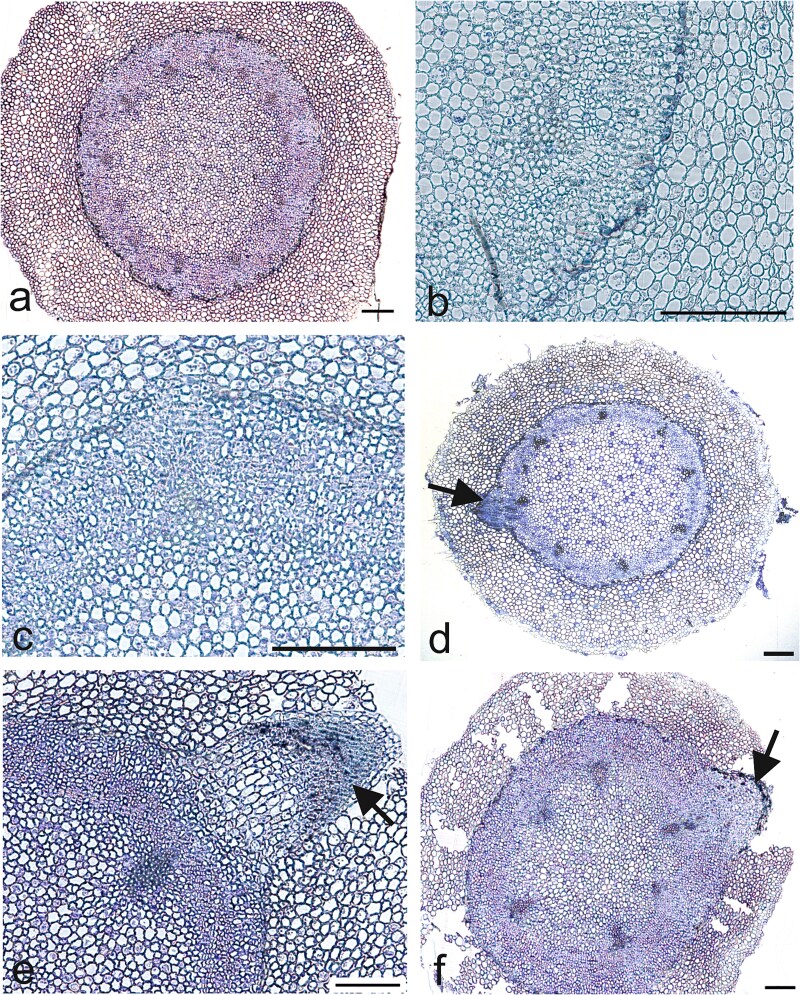
Transverse sections of lateral root formation and emergence (arrows) within the maturation zone of short (a, b), medium (c, d) and long (e, f) taproot classes. Scale bars = 200 μm.

In the differentiation zone, all stages of histogenesis were observed in root cross sections, starting from the vascular cylinder, distinguished by the maturation of the first protophloem elements, to the first primary phloem and xylem strands. Subsequently, metaxylem matures, and the stele consists of numerous primary phloem and xylem strands. This type of root is polyarch, with 6–10 vascular strands (Figure 4). Moreover, the emergence of lateral root was visible when the tap root reached an average length 9 cm, while the profound growth of lateral roots occurred in long taproot length class (>15.5 cm; Figure 4).

### Gene expression level changes within roots during elongation

Recognition of genes regulated growth and development of taproots and lateral roots, gene expression profiles was analyzed using RNA-Seq analysis. Only records with log2FC < −1.5 or log2FC > 1.5 were used in the analyses of up-regulated and down-regulated differentially expressed genes. Furthermore, comparing the sequenced transcriptomes allowed us to identify genes engaged in the development of both root types. We compared transcriptomes of taproots and emerging lateral roots to determine the genetic landscape at different stages of root elongation DEGs analysis was performed for the taproot compared with the lateral root. However, if we were to analyze the number of DEGs in the second comparison, the analysis would be inverted. The number of down-regulated genes in the first variant corresponds to the number of up-regulated genes in the second comparison, and conversely, the number of up-regulated genes in the first variant corresponds to the number of down-regulated genes in the second variant. In this work, we aim to identify DEGs associated with plant hormones, recognizing their crucial role in regulating root growth (Table S3 available as Supplementary Data at *TreePhysiology* Online). Additionally, we examine transcription factors that may interact with these hormonal pathways to better understand the regulatory networks governing taproot development in oak.

**Figure 5:**
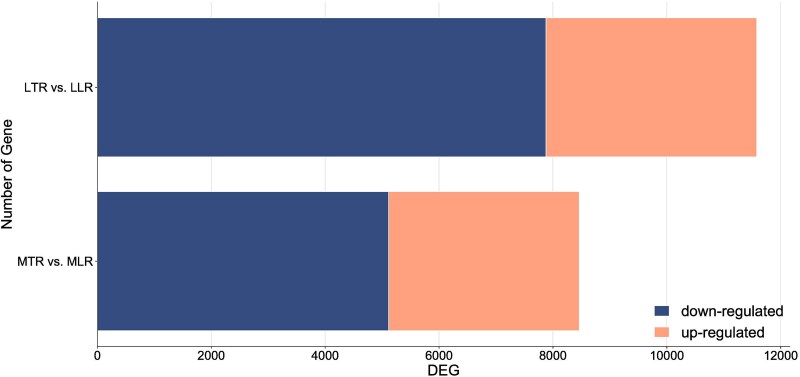
Number of differential DEGs between taproot and lateral root at different stages of growth. LTR—long taproot characterized by standard morphology; LLR—lateral roots harvested from a long taproot of standard morphology; MTR—medium taproot characterized by standard morphology; MLR—lateral roots harvested from a medium taproot of standard morphology.

**Figure 6:**
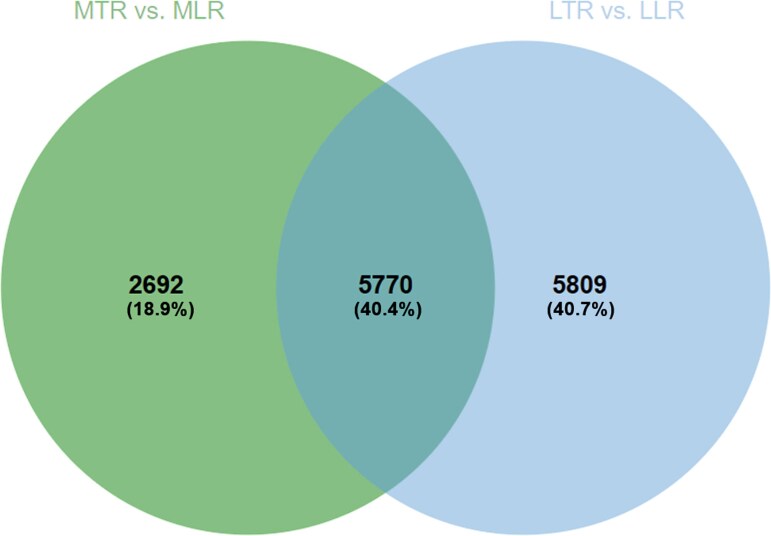
Venn diagram showing the number of genes mutually expressed in the medium taproot (MTR) and their lateral roots (MLR), i.e., 2692, or in the long taproot (LTR) and their lateral roots (LLR), i.e., 5809, and those common to both taproot length classes, i.e., 5770.

Evaluation of DEG expression revealed that 20,041 DEGs exhibited differential expression patterns between different root types (in medium and long roots) (Figure 5), including 12,993 down-regulated and 7048 up-regulated genes in taproot compared with lateral roots (Figure 5). This may indicate the activation of other genes during the elongation of the taproot and lateral roots, as suggested by differences in gene expression in the elongating root. When the roots reached medium length, the number of down-regulated genes was 5115 and up-regulated genes 3347 in lateral roots compared with the taproot (Figure 5). An even greater difference in gene expression occurred when comparing LTR and their lateral roots (Figure 5; 7878 down-regulated and 3701 up-regulated in lateral roots compared with the taproot). At this elongation points the taproots showed more genes with reduced expression relative to the lateral roots, suggesting elevated genes activation in lateral roots (Table S1 available as Supplementary Data at *TreePhysiology* Online). The number of differentially expressed genes contrasting taproot to lateral roots increased from 2692 (MTR vs MLR) to 5809 (LTR vs long lateral roots—LLR) as the taproots elongated, with 5478 differentially expressed genes common to both comparisons (Figure 6). All genes exhibiting differential expression are presented in Table S1 available as Supplementary Data at *TreePhysiology* Online.

We also investigated whether the expression levels of hormone-related genes varied based on root type. Differential expression gene analysis for medium and long taproot apices in comparison with lateral roots derived from both MTR or LTR, respectively, revealed similar trends in up- and down-regulation of genes associated with the biosynthesis of hormones in both comparison (Table 1 and Table S3 available as Supplementary Data at *TreePhysiology* Online). Among signal transduction and response pathway genes, DEGs related to all hormones, except for jasmonic acid (JA), was identified. The highest number of DEGs in this category were associated with auxin (IAA, ABA and ET).

**Table 1:** Differentially expressed genes (DEGs) involved in plant hormone metabolic and signaling pathways in taproot (MTR vs LTR) and lateral root (MLR vs LLR) comparisons. IAA—indole-3-acetic acid; CK—cytokinins; ABA—abscisic acid; ET—ethylene; JA—jasmonic acid; GA—gibberellins; BR—brassinosteroids.

MTR vs MLR				LTR vs LLR			
Hormone	Total no. of DEGs	Down-regulated	Up-regulated	Total no. of DEGs	Down-regulated	Up-regulated	Function
IAA	27	14	13	29	20	9	Signal transduction
	9	5	4	13	10	3	Transport
	4	2	2	6	2	4	Biosynthesis
	5	4	1	2	2	–	Conjugate synthesis
	2	2	–	2	2	–	Conjugate degradation
	4	3	1	4	4	–	Degradation/inactivation
CK	2	2	–	4	2	2	Biosynthesis
	2	2	–	3	3	–	Signal transduction
	1	1	–	1	1	–	Conjugate synthesis
ABA	27	17	10	41	34	7	Signal transduction
	2	1	1	3	3	–	Biosynthesis
	1	1	–	2	2	–	Degradation/inactivation
ET	26	14	12	25	15	10	Signal transduction
	8	4	4	13	10	3	Biosynthesis
JA	9	7	2	14	10	4	Biosynthesis
	1	1	–	5	4	1	Degradation/inactivation
	–	–	–	1	1	–	Conjugate synthesis
GA	6	3	3	2	1	1	Biosynthesis
	6	3	3	4	3	1	Signal transduction
	2	2	–	1	1	–	Degradation/inactivation
BR	10	7	3	7	5	2	Signal transduction
	4	4	–	5	4	1	Degradation/inactivation
	3	–	3	3	–	3	Biosynthesis

### Functional annotation—GO and KEGG analysis

The selected genes were characterized by GO terms. Genes exhibiting increased expression comparing medium taproots and their lateral roots (MTR vs MLR) and long taproot and their lateral roots (LTR vs LLR) were subsequently classified using a set of plant-specific GOs and grouped into following categories: biological process (BP), cellular component (CC) and molecular function (MF).

**Figure 7:**
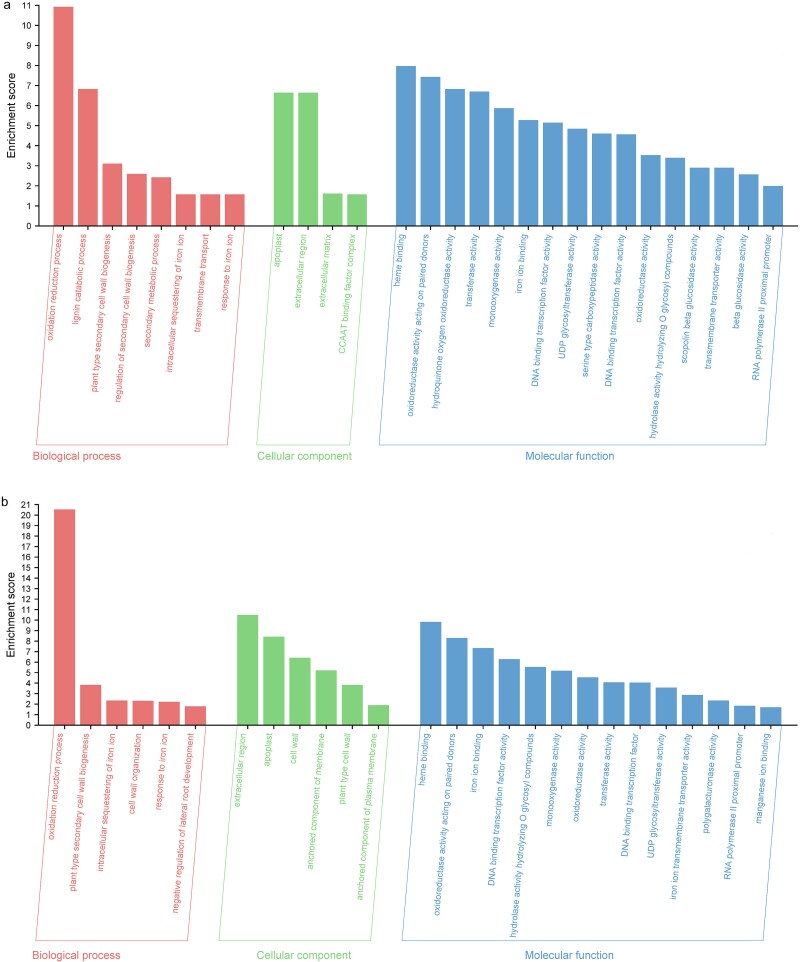
Gene Ontology (GO) term enrichment analysis between (a) medium taproots and their lateral roots, (b) long taproot and their lateral roots. The GO terms that were significantly enriched were chosen based on a false discovery rate (FDR) of < 1.5. Enrichment scores represent −log10(FDR).

Detailed results of the GO analysis for differentially DEGs comparing medium taproots and their lateral roots (MTR vs MLR; Figure 7a,) and long taproot and their lateral roots (LTR vs LLR; Figure 7b). In medium-length taproots and their laterals, the most enriched genes were related to oxidation–reduction processes (BP), apoplast and extracellular region (CC) and heme binding (MF) (Figure 7a), while in long taproots and their laterals, they were related to oxidation–reduction processes (BP), extracellular region (CC) and heme binding (MF) (Figure 7b). Although in the BP category, genes associated with the oxidation–reduction process were the most enriched when comparing medium and long roots, it is worth noting that in long roots, this increase was much greater than in medium roots (⁓21 vs 11). Similarly to other terms, enrichment is higher in long roots than in medium roots.

**Figure 8:**
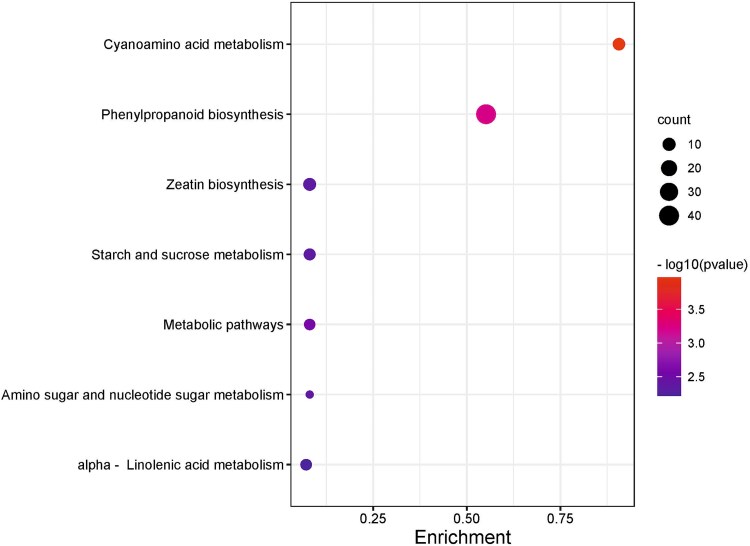
KEGG enrichment analysis between taproot and their lateral roots. The figure displays KEGG metabolic pathways, where each circle represents a pathway, and the size of the circle corresponds to the number of genes enriched in that pathway. The degree of significance of the enrichment of differentially expressed genes (DEGs) in a pathway is indicated by −log10 (*P*-value). Low q-values are below 2.5, and high q-values are above 3.5; the size of the circle is proportional to the number of enriched genes.

To gain further insights into metabolic pathways and signal transduction, we conducted a (KEGG) pathway analysis of DEGs. The most significantly enriched KEGG pathways between taproot and lateral roots are displayed in Figure 8. Among them, the highest levels of significance were observed for cyanoamino acid metabolism, phenylpropanoid biosynthesis and metabolic pathways.

### Validation of the RNA-Seq results

**Figure 9:**
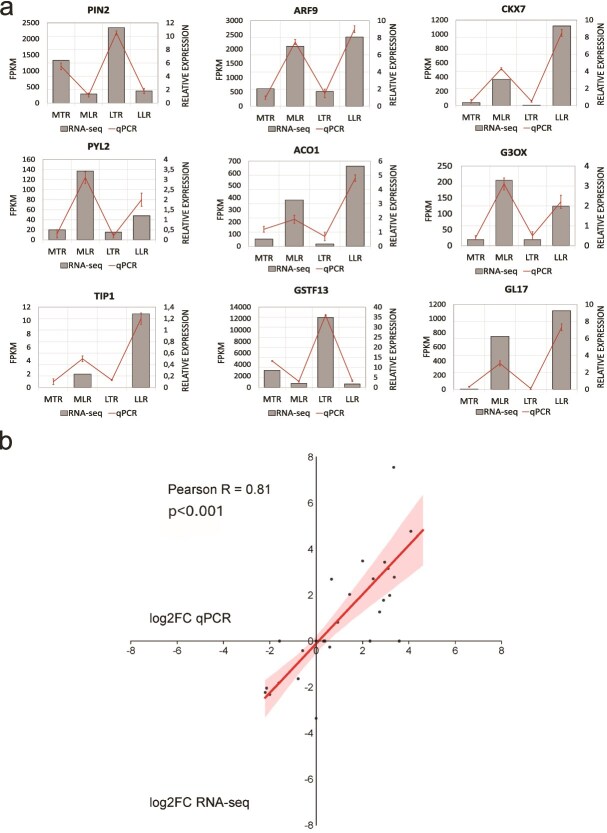
Correlation between gene expression levels obtained from RNA-Seq and RT-qPCR analyses. (a) Graphical representation demonstrating consistent expression trends for individual genes as determined by both RNA-Seq and RT-qPCR; the bar chart presents RNA-Seq data from a single sample for each analyzed variant. The corresponding line graph displays the RNA-Seq validation results obtained by RT-qPCR. For RT-qPCR analysis, the same samples used for RNA-Seq were measured in three technical replicates for each analyzed gene. The error bars represent the standard error of the mean calculated from the RT-qPCR technical replicates. (b) Pearson correlation analysis based on log2FC values, comparing gene expression levels derived from RNA-Seq with those quantified by RT-qPCR. The correlation plot highlights the strong agreement between the two methodologies. *PIN2*—*Auxin efflux carrier component 2*; *ARF9—Auxin response factor 9*; *CKX7—Cytokinin oxidase 7*; *PYL2—Abscisic acid receptor PYL2*; *ACO1–1-aminocyclopropane-1-carboxylate oxidase homolog 1*; *G3OX—Gibberellin 3-beta-dioxygenase 1*; *TIP1—Aquaporin TIP1*; *GSTF13—Glutathione s-transferase f13*; *GL17—Germin-like protein subfamily 1 member 7*.

We evaluated the expression of nine genes for each experimental variant (MTR, MLR, LTR, LLR) to validate the gene expression results obtained from RNA-Seq analysis. Given the focus of our study on hormone-related genes, we selected six genes associated with hormone pathways (*PIN2*, *ARF9*, *CKX7*, *PYL2*, *ACO1*, *G3OX*) and three genes crucial for root growth and development (*TIP1*, *GSTF13*, *GL13*) for validation. The RT-qPCR analysis revealed a strong concordance in the expression levels of these genes compared with the RNA-Seq data (Figure 9a). Furthermore, Pearson correlation analysis, with an R^2^ = 0.81, confirmed a high degree of correlation between the RNA-Seq and RT-qPCR results (Figure 9b). These findings underscore the reliability and consistency of our transcriptomic data.

### The effect of hormones on root morphology

**Figure 10:**
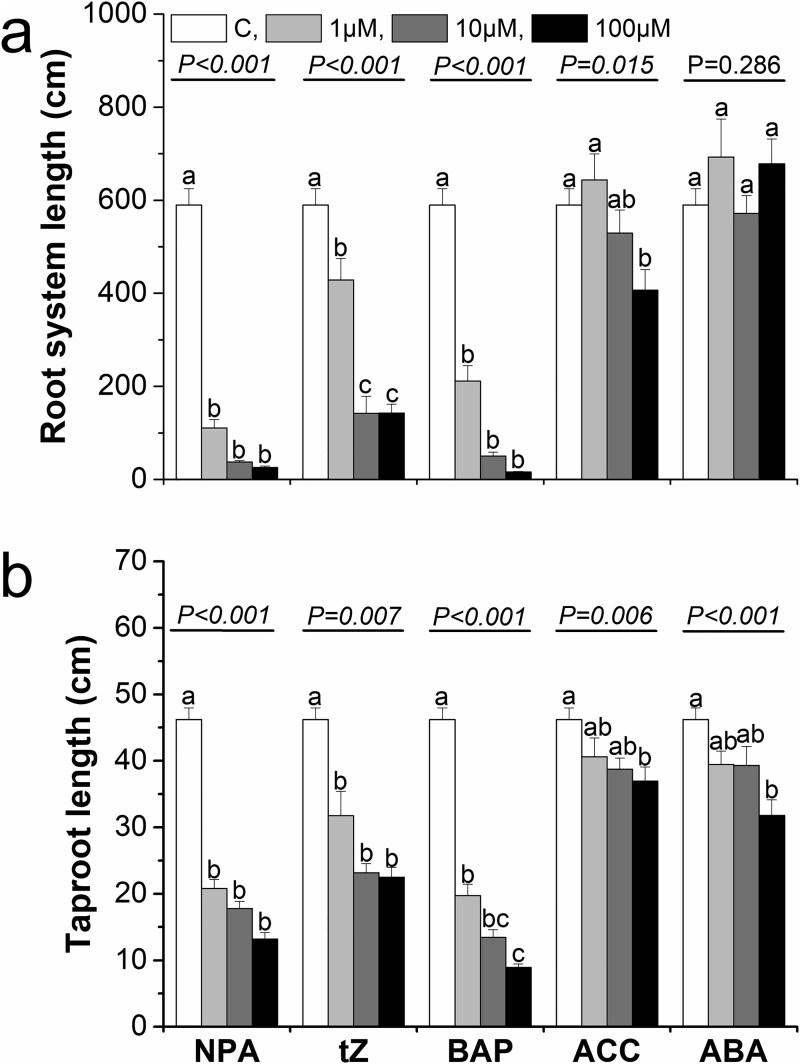
The effect of hormone application on the root system length (a) and taproot length (b) when applying NPA, tZ, BAP, ACC, ABA at control conditions (open columns) and different hormone concentrations: 1 μM (light gray columns), 10 μM (heavy gray columns) and 100 μM (black columns). Different lowercase letters indicate significant differences among concentration treatments within each hormone application (α = 0.05), according to Tukey’s HSD test (*P*-value from the ANOVA for hormone concentration treatment is shown above the line). Each treatment included three replications, with 10 seedlings analyzed per biological replicate. Error bars represent the standard error. NPA—naphthylphthalamic acid; tZ—trans-zeatin; BAP—6-benzylaminopurine; ABA—abscisic acid; ACC—1-aminocyclopropane-1-carboxylic acid.

**Figure 11:**
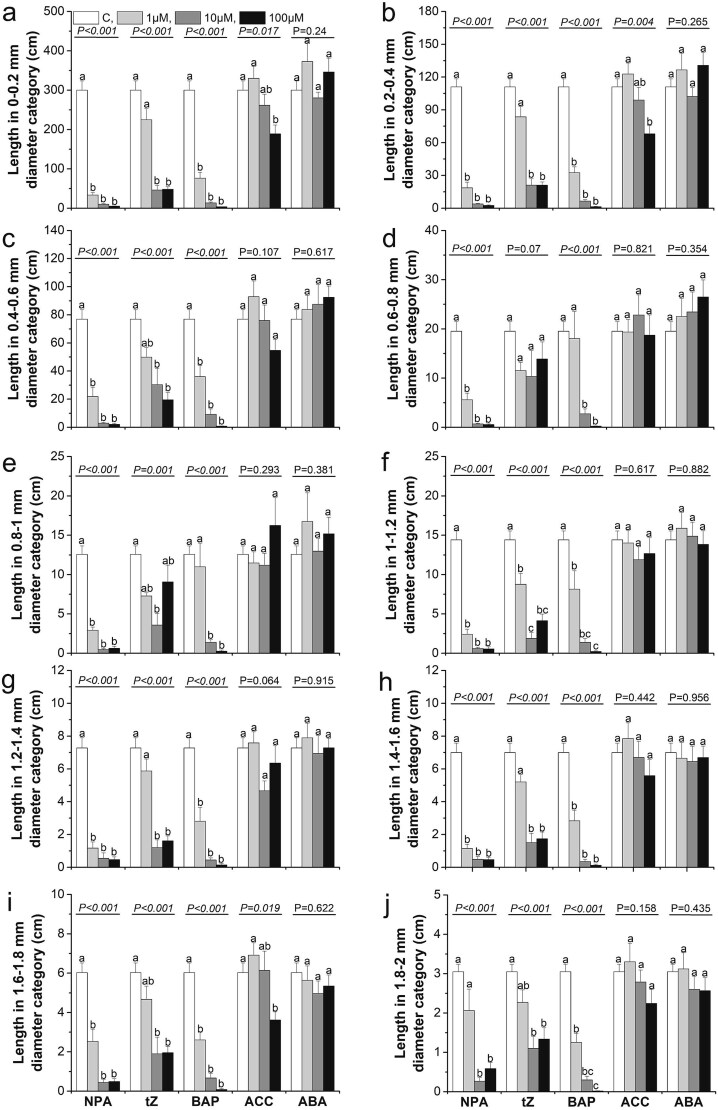
The effect of hormone application on root length within given diameter categories: 0–0.2 mm (a), 0.2–0.4 mm (b), 0.4–0.6 mm (c), 0.6–0.8 mm (d), 0.8–1 mm (e), 1–1.2 mm (f), 1.2–1.4 mm (g), 1.4–1.6 mm (h), 1.6–1.8 mm (i) and 1.8–2 mm (j), when applying NPA, tZ, BAP, ACC, ABA at control conditions (open columns) and different hormone concentrations: 1 μM (light gray columns) 10 μM (heavy gray columns) and 100 μM (black columns). Different lowercase letters indicate significant differences among concentration treatments within each hormone application (α = 0.05), according to Tukey’s HSD test (*P*-value from the ANOVA for hormone concentration treatment is shown above the line). Each treatment included three replications, with 10 seedlings analyzed per biological replicate. Error bars represent the standard error. NPA—naphthylphthalamic acid; tZ—trans-zeatin; BAP—6-benzylaminopurine; ABA—abscisic acid; ACC—1-aminocyclopropane-1-carboxylic acid.

**Figure 12:**
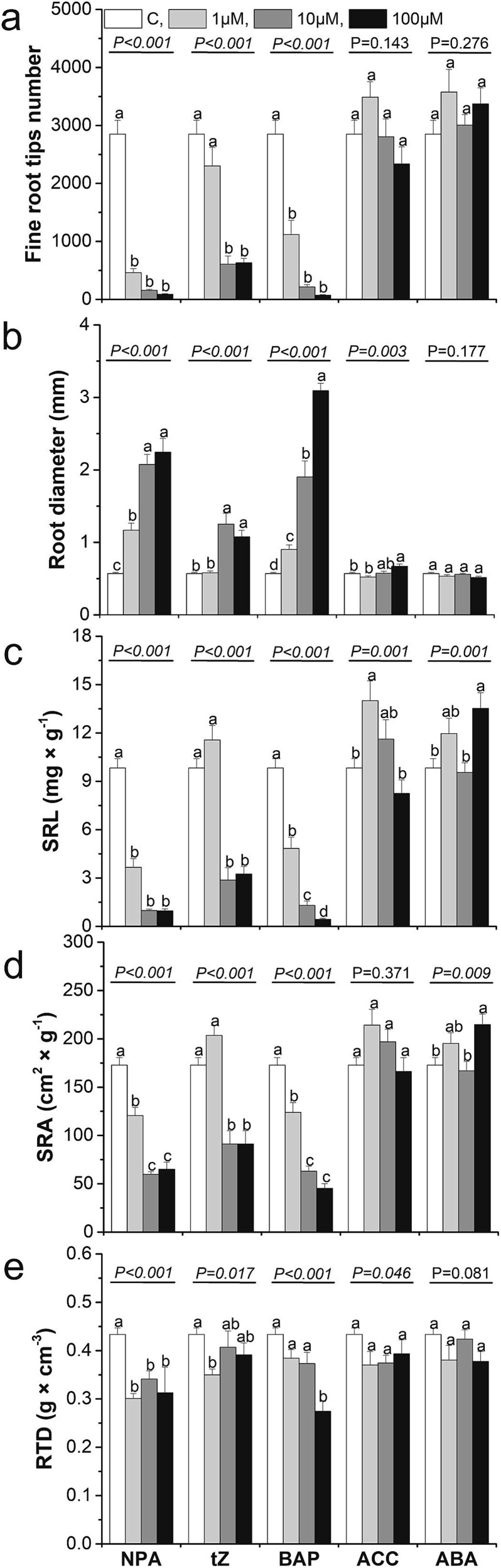
The effect of hormone application on number of fine root tips per root system (a), root diameter (b), specific root length-SRL (c), specific root area-SRA (d) and root tissue density-RTD (e) when applying NPA, tZ, BAP, ACC, ABA at control conditions (open columns) and different hormone concentrations: 1 μM (light gray columns), 10 μM (heavy gray columns) and 100 μM (black columns). Different lowercase letters indicate significant differences among concentration treatments within each hormone application (α = 0.05), according to Tukey’s HSD test (*P*-value from the ANOVA for hormone concentration treatment is shown above the line). Each treatment included three replications, with 10 seedlings analyzed per biological replicate. Error bars represent the standard error. NPA—naphthylphthalamic acid; tZ—trans-zeatin; BAP—6-benzylaminopurine; ABA—abscisic acid; ACC—1-aminocyclopropane-1-carboxylic acid.

Since transcriptomic analysis revealed that hormones play a significant role in regulating morphology and the formation of lateral roots, we decided to investigate their effects using exogenous hormone treatments in hydroponic experiments. For this study, we selected an auxin transport inhibitor (due to the substantial number of genes involved in IAA transport and signal transduction) as well as the hormones CK, ABA and ET (due to the substantial number of genes encoding these hormones identified in the DEG analysis). Given the challenges of applying ET in its gaseous form, we used its precursor, ACC (1-aminocyclopropane-1-carboxylate), instead. In our study, we observed significant differences among the applied hormones with respect to their effects on the analyzed root traits (Table S4 available as Supplementary Data at *TreePhysiology* Online). We observed a trend that application of exogenous NPA (an auxin inhibitor, N-1-naphthylphthalamic acid) significantly reduced values of many analyzed traits, especially taproot length, length of the lateral roots (especially in the most absorptive root diameter categories i.e., 0–0.6 mm) and most morphological root traits associated with resource acquisition (no. of tips, SRL, SRA) irrespectively of applied hormone concentrations (Figures 10–12). Similar pattern was observed after both cytokinin (tZ and BAP) application (Figures 10–12). Seedlings' traits response after application of other hormones are more variable and more depended on the used concentration, e.g., root length reduction after ACC (precursor of ethylene) application was highest at the 100 μM concentration (Figures 10–12).

## Discussion

Although the importance of primary roots meristem in lateral root development has been investigated previously, to our knowledge, little is known about molecular basis of trees taproot and lateral roots growth (Waidmann et al. 2020). Accordingly, our approach aimed to understand the molecular basis that control the growth of taproots and emergence of lateral roots, as well as the transcriptional characteristics at key stages of the former elongation. This enhances our knowledge of root development at the whole-genome level. Therefore, we analyzed gene expression to characterize global transcript changes during taproot and lateral root organogenesis. Our findings revealed that the gene expression profile during taproot elongation is linked to the emergence of their lateral roots.

### Regulation of lateral roots formation by taproots

There was a significant dependence between taproot length classes and the observed gene expression profiles. The longer the root, the more genes were activated, and an increasing number of genes showed higher expression in lateral roots compared with taproots. Taproot elongation, sensu stricto, is regulated by a relatively low number of genes. When comparing DEGs expression between taproots and their laterals, we observed a higher number of up-regulated genes and fewer downregulated genes in the lateral roots, both in medium and long roots. This indicates that the emergence from specific taproot length classes determines the genes profile of lateral roots, but the elongation of lateral roots itself also requires other genes than those regulating taproot growth. This gene expression pattern confirms a distinction between lateral roots produced early, soon after taproot emergence, and those that appear later when the taproot is longer.

Primary root growth and lateral root formation is interrelated on the level of specific gene expression. According to apical dominance paradigm, primary roots ‘coordinates’ lateral root emergence and growth. Indeed, in our study, we observed elevated levels of genes involved in biosynthesis of linoleic acid in taproots (both medium and long), which serves as a precursor of JA. Jasmonic acid inhibits primary root growth while promoting lateral root growth (Huang et al. 2017). This was consistent with the elevated expression of the *MYB93* gene in medium and long taproots (Table S1 available as Supplementary Data at *TreePhysiology* Online), a gene involved in lateral root initiation in Arabidopsis, as previously demonstrated by Uemura et al. (2022). Additional evidence for the crucial role of taproots in lateral root initation was provided by an increased MAPK expression (Mitogen-activated protein kinase; KEGG analyses—Figure 8), which through auxin mediated biosynthesis of long-chain fatty acids regulates lateral roots emergance (Huang et al. 2017).

Indeed, medium and long lateral roots exhibited higher expression of the genes encoding the *Auxin-response factor* (*ARF*) and *Auxin-response protein* (*SAUR*) (Table S1 available as Supplementary Data at *TreePhysiology* Online), which are directly involved in coordinating lateral root length, controlling cell division, and differentiating vascular cambium by regulating auxin or gibberellin concentration, as demonstrated in various plants including wild and mutant *Medicago truncatula* or *Populus* (Etchells et al. 2013, Kucukoglu et al. 2017). ARF proteins are also involved in auxin/indole-3-acetic acid (Aux/IAA) modules mediating lateral root founder cell initiation, specification, and formation in Arabidopsis (Goh et al. 2012). The increased expression of these genes in our results suggests that the primary root initiates lateral root growth. Enhanced expression of *MYC2*-JA response genes may further confirm that the medium length of taproot is the point of regulation for the lateral root formation, corresponding to their role in controlling adventitious and lateral root development (reviewed in Browse (2009).

Possibly, the identified genes perform similar functions during oak root development. Moreover, the observed increases zeatin biosynthesis (KEGG pathways, Figure 8) should be linked with inhibition of lateral root development in GO analysis (Figure 7) as a results of its antagonism against auxin (Fukaki and Tasaka 2009, Peres et al. 2019). The expression of *CKX* within MTR may enable oaks to regulate the apical dominance of primary roots, causing lateral roots to emerge and grow at a greater distance from the root tip as was shown in Arabidopsis by Aloni et al. (2006). Our findings demonstrate that the inhibition of lateral root emergence and the reduction in root length, observed following the application of auxin transport inhibitors or cytokinins (BAP and tZ; Figure 10 and Figure S1 available as Supplementary Data at *Tree Physiology* Online) in hydroponic conditions, confirm the essential role of auxin-mediated pathways in regulating lateral root development. Additionally, we observed that the expression patterns of genes related in MAPK pathway and *LRP1* genes correlated with lateral root emergence from taproots (Figure 5, Table S1 available as Supplementary Data at *TreePhysiology* Online). Further support that in long taproots the processes related to emergence of lateral roots was provided by increased expression of gene coding TF KUA1 through auxin accumulation in a phytochrome-interacting factor (PIF) proteins-dependent manner (Kwon et al. 2013). Transcription factor KUA1 expression suggests that the elongation engaged the same mechanism irrespectively root or hypocotyl growth.

Indeed, after lateral roots emergence and growth is initiated and proceed, molecular machinery in tips of taproot focuses on the their elongation. Taproot elongation appears to be sequentially regulated by MYB111, which showed elevated expression in LTR. This transcription factor likely modulates gibberellin signaling pathways that interact with auxin (IAA) pathways, as suggested by Tan et al. (2019). Additionally, enhanced expression of MYB2 and WER together with CPC occurred mainly in long taproots, and led to the formation of a three-component complex may regulating root cell differentiation in these root classes (Table S1 available as Supplementary Data at *TreePhysiology* Online) (Chen et al. 2022).

### Variation of lateral roots gene expression pattern

We have discovered that both MLR and LLR show lower expression of genes related to brassinosteroids biosynthesis (Table 1). This pattern of BR biosynthesis gene expression suggests that during the initial stages of taproot growth in species such as oaks, which are characterized by large reserves accumulated in acorns, priority is given to deep soil exploration by rapidly growing taproots at the expense of lateral root growth. The observed higher expression of BR biosynthesis genes in root tips of taproots, together with genes encoding CK, may further underline the apical dominance of taproot tips, resulting in their elongation to accelerate water uptake, as shown for lateral roots by (Bao et al. 2004, Fàbregas et al. 2018, Kościelniak et al. 2021, Vukašinović et al. 2021). Further confirmation is needed to verify the role of brassinosteroids in promoting oak taproot growth, specifically through studies using brassinosteroid-deficient mutants. On the other hand, enhanced expression of the *CKX* gene, encoding the cytokinin-degrading enzyme cytokinin oxidase, in lateral roots suggests functional root type diversification of CK biosynthesis (Figure 9). This diversification, on one hand, allows lateral root development by removing the cytokinin signal (Kuroha et al. 2009, Del Bianco et al. 2013), and on the other hand, enables CK-related apical dominance of the taproot. We indicated that application of tZ or BAP stronger limited finest root length (0–0.6 mm category) (Figures 10 and 11). The presence of auxin-induced negative regulators of lateral root development and growth, such as *MYB93*, may generally explain why lateral roots within MTR have lower growth potential (Gibbs et al. 2014). Conversely, a higher presence of *MYB2* and *WER* in LTR may promote lateral root development and induce root hair formation within them, as demonstrated by (Chen et al. 2022). Within lateral roots emerged either on MTR or LTR, i.e., medium (MLR) and long lateral roots (LLR), growth occurs concomitantly with enhanced accumulation of genes coding TFs NFYB4, MOF1, TCP2 and ORG2 among which the first is upregulated in both leaves and roots (Wang et al. 2021). Although their roles in lateral root growth are not yet known, considering the large number of transcripts, it seems that they are necessary for lateral root initiation and growth. This indicates strict coordination of transcription factors among relatively short but distinct points during taproot elongation. Evidence for the time needed for lateral roots to acquire functionality is shown by the higher expression level of *WRK51* observed in both medium and long lateral roots (MTR and LTR). This gene promoting lateral root emergence and growth by inhibition of ethylene biosynthesis in wheat (*Triticum aestivum* L.) has been shown by (Hu et al. 2018). Overexpression of the same gene (**Ta*WRKY51*) and increased expression of NAC56 and RAX3 resulted in more crown and lateral roots in rice, of which increased expression of these genes was observed in MLR and LLR promoting lateral root growth and controlling shoot branching in *Arabidopsis thaliana* (Guo et al. 2015, Xu et al. 2022), suggesting that their expression can be an important regulator of lateral root branching. On the other hand, changes at the level of genes encoding proteins involved in phytohormone biosynthesis, affecting GA (G3OX and G20X2), ABA (NCED1) and most notably JA (LOX and OPR family genes), could explain that lateral root harvested from MTR may differ from lateral roots growing on LTR. Our research shows how important it is to locate the collected material in time and growth stage when interpreting gene functionality.

## Conclusions

Our study provided insight into the connection between gene patterning related to root elongation, taproot organogenesis mechanisms and the regulation of lateral root development. We indicated that growth trade-off between taproot elongation and lateral root emergence may be linked to genes coding for transcription factors such as *bHLH12*, *TCP15* and *WRKY75*. These exhibit greater expression in lateral roots emergence, particularly in the MTR. The patterning of lateral roots emergence involves interactions among auxin and MAPK, suggesting that this regulation is a common process. Indeed, in our study, lateral roots showed increased expression of genes involved cytokinin-degrading enzyme encoding the CKX gene. The importance of cytokinin inhibition as a way that control lateral roots emergence was further confirmed by application of tZ and BAP in hydroponic experiment, with gene expression levels validated by RT-qPCR analysis. The overall patterns of lateral root development studied here indicated strong relation to taproot elongation. Given the striking differences in development observed in the taproot and lateral roots, it is clear that quantitative transcriptome appreciation of interspecific variation both root types is needed to understand oaks adjustment to develop a deep root system. Furthermore, the observed patterns, together with the comprehensive functional annotation of genes, suggest partial hormonal regulation. This is supported by results from the hydroponic experiment, which indicate changes in root morphological and structural traits during oak taproot system formation in response to internal signals. This knowledge has significant practical implications for improving forest management operations, particularly in seedling regeneration.

## Supplementary Material

Figure_S1_tpaf067

Supplementary_materials_tpaf067_Table1

Supplementary_materials_tpaf067_Table2

Supplementary_materials_tpaf067_Table3

Supplementary_materials_tpaf067_Table4

## Data Availability

The data underlying this article are available in the NCBI GEO Database at https://www.ncbi.nlm.nih.gov/geo/query/acc.cgi?acc=GSM5513288 and can be accessed with accession number GSE181860. The data underlying this article are available in the article and in its online supplementary material.
