# Supplementary material for: Comparative RNA sequencing-based transcriptome profiling of Quercur robur: specific sets of genes involved in taproot and lateral roots emergence
Source: Tree Physiol. 2025 Jun 2;45(6):tpaf067. doi: 10.1093/treephys/tpaf067 (PMC12207064; doi:10.1093/treephys/tpaf067)
Supplement: Figure_S1_tpaf067 [file figure_s1_tpaf067.docx]

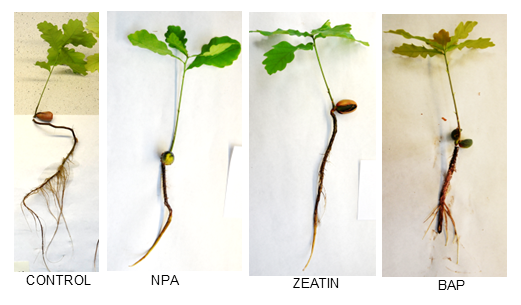


Figure S1. The effect of inhibitor (NPA - naphthylphthalamic acid) and hormone applications (trans-Zeatin; BAP - 6-Benzylaminopurine) on the morphology of *Quercus robur* root system.
