# Supplementary material for: Comparative RNA sequencing-based transcriptome profiling of Quercur robur: specific sets of genes involved in taproot and lateral roots emergence
Source: Tree Physiol. 2025 Jun 2;45(6):tpaf067. doi: 10.1093/treephys/tpaf067 (PMC12207064; doi:10.1093/treephys/tpaf067)
Supplement: Supplementary_materials_tpaf067_Table4 [file supplementary_materials_tpaf067_table4.docx]

**Supplementary Table 4.** Results of analysis of variance (one-way ANNOVA) of analyzed hormones and control conditions in hydroponic experiment (H) on A) root system length (System), taproot length (Taproot), root length in 0-0.2 mm diameter category (0-0.2), root length in 0.2-0.4 mm diameter category (0.2-0.4), root length in 0.4-0.6 mm diameter category (0.4-0.6), root length in 0.6-0.8 mm diameter category (0.6-0.8), root length in 0.8-1 mm diameter category (0.8-1), root length in 1-1.2 mm diameter category (1-1.2); B) root length in 1.2-1.4 mm diameter category (1.2-1.4), root length in 1.4-1.6 mm diameter category (1.4-1.6), root length in 1.6-1.8 mm diameter category (1.6-1.8), root length in 1.8-2 mm diameter category (1.8-2); C) fine root tips per root system (Fine root tips number), mean root diameter (Root diameter), specific root length (SRL), specific root area (SRA) and root tissue density (RTD).

| Characteristic | |  | d.f. | | *F* | | *P* | *F* | | | | *P* | | *F* | | *P* |  | | *F* | | | *P* | | *F* | | *P* | | *F* | | *P* | |  |
| --- | --- | --- | --- | --- | --- | --- | --- | --- | --- | --- | --- | --- | --- | --- | --- | --- | --- | --- | --- | --- | --- | --- | --- | --- | --- | --- | --- | --- | --- | --- | --- | --- |
| *A) Root length production* | Source |  | System | | | | |  | Taproot | | | |  | 0-0.2 | | |  | 0.2-0.4 | | | | | |  | 0.4-0.6 | | | | 0.6-0.8 | | | |
|  | H |  | 5 | 75.31 | | *<0.001* | |  | 79.81 | *<0.001* | | |  | 61.62 | *<0.001* | |  | 65.83 | | *<0.001* | | | |  | 38.33 | | *<0.001* | | 18.42 | | *<0.001* | |
| *B) Root length production* |  |  |  | 0.8-1 | | | |  | 1-1.2 | | | |  | 1.2-1.4 | | |  | 1.4-1.6 | | | | | |  | 1.6-1.8 | | | | 1.8-2 | | | |
|  | H |  | 5 | 20.82 | | *<0.001* | |  | 40.73 | | *<0.001* | |  | 41.08 | *<0.001* | |  | 39.5 | | | | | *<0.001* |  | 31.41 | | *<0.001* | | 26.91 | | *<0.001* | |
| *C) Root morphological traits* |  |  |  | Fine root tips number | | | |  | Root diameter | | | |  | SRL | | |  | SRA | | | | | |  | RTD | | | |  | |  | |
|  | H |  | 5 | 60.9 | | *<0.001* | |  | 55.4 | | *<0.001* | |  | 50.52 | *<0.001* | |  | 39.66 | | | *<0.001* | | |  | 7.1 | | *<0.001* | |  | |  | |

Italics indicate significant effect at *P*≤0.05.

d.f. = degrees of freedom, *P* = P value, *F* = F ratio
